# Exercise limitation in chronic kidney disease: An experimental pilot study with leg and arm exercise

**DOI:** 10.14814/phy2.70200

**Published:** 2025-01-14

**Authors:** Helena Wallin, Eva Jansson, Ragad Said, Sigrid Lundberg, Pourya Zolfaghardidani, Maria J. Eriksson, Anette Rickenlund, Patrik Sundblad

**Affiliations:** ^1^ Division of Clinical Physiology Department of Laboratory Medicine Karolinska Institutet Stockholm Sweden; ^2^ Department of Clinical Physiology Karolinska University Hospital Stockholm Sweden; ^3^ Nephrology Clinic Danderyds Hospital Stockholm Sweden; ^4^ Department of Clinical Science Danderyds Hospital, Karolinska Institutet Stockholm Sweden; ^5^ Department of Clinical Science and Education Södersjukhuset, Karolinska Institutet Stockholm Sweden; ^6^ Department of Molecular Medicine and Surgery Karolinska Institutet Stockholm Sweden

**Keywords:** arm exercise, exercise capacity, exercise limitation, kidney disease

## Abstract

Maximal oxygen uptake (VO_2_max) in healthy subjects is primarily limited by systemic oxygen delivery. In chronic kidney disease (CKD), VO_2_max is potentially reduced by both central and peripheral factors. We aimed to investigate the effect on VO_2_peak of adding arm exercise to leg exercise. Ten individuals with CKD stages 3–5 and 10 healthy controls, matched for age, sex, body size, and physical activity level, were included. Subjects performed two maximal exercise tests, one with legs only (L exercise) and one test where arm exercise was added to leg exercise (LA exercise). The increase in VO_2_peak, when comparing LA exercise with L exercise, was significantly higher in CKD (0.20 ± 0.18 L/min or 2.31 ± 1.78 mL/(kg·min)) than in controls (0.019 ± 0.12 L/min or 0.26 ± 1.62 mL/(kg·min); *p* = 0.02 and 0.01, respectively). The decrease in peak leg workload, when comparing L exercise with LA exercise, was larger in controls than in CKD, in absolute terms (*p* = 0.002) and relative to body weight (*p* = 0.01). VO_2_max in individuals with CKD is dependent on the active muscle mass, supporting a peripheral limitation to VO_2_max in CKD. By contrast, the control group appeared to have a more central limitation to VO_2_max.

## 1 INTRODUCTION

Aerobic exercise capacity, often measured as maximal oxygen uptake (VO_2_max), is reduced in chronic kidney disease (CKD) and decreases gradually with disease severity (Faria Rde et al., 2013; Howden et al., 2015; Painter et al., 1986; Wallin et al., 2018). As reduced exercise capacity is associated with worse quality of life (Lopes et al., 2014) and increased mortality (Sietsema et al., 2004), a better understanding of the mechanisms contributing to exercise intolerance in CKD is important.

VO_2_max is a function of oxygen delivery and utilization. In healthy individuals, VO_2_max is thought to be limited by systemic oxygen delivery, which in turn is mainly limited by stroke volume (Bassett Jr. & Howley, 2000; Levine, 2008). The cause behind a reduced VO_2_max in individuals with CKD is likely multifactorial. Systemic oxygen delivery factors contribute to explain the variation in aerobic exercise capacity also in CKD, but besides stroke volume, hemoglobin (Hb) level and peak heart rate seem to be important determinants (Chinnappa et al., 2018; Howden et al., 2015; Odden et al., 2004; Wallin et al., 2018). In addition to central factors influencing systemic oxygen delivery, peripheral factors may limit VO_2_max in CKD. The peripheral changes in CKD encompass both circulatory and structural changes in the muscle. Impaired muscle blood flow (Bradley et al., 1990; Sprick et al., 2019), a low capillary density (Moore, Parsons, et al., 1993) and a thickened capillary membrane (Stray‐Gundersen et al., 2016) could potentially reduce peripheral oxygen delivery. Moreover, reduced muscle mass (Isoyama et al., 2014) and mitochondrial dysfunction (Gamboa et al., 2016; Kestenbaum et al., 2020; Roshanravan et al., 2016; Sakkas et al., 2003) have been reported in CKD and may limit oxygen utilization.

To reach VO_2_max, the working muscle mass needs to be large enough to tax the heart and aerobic system to its maximum (Rowell, 1974). In most individuals, the work performed during upright bicycling with traditional leg exercise is enough to elicit a true VO_2_max (Astrand & Saltin, 1961; Secher et al., 1974), but this may not be the case in individuals with CKD because of peripheral circulatory and muscular factors limiting VO_2_max. To study the influence of the working muscle mass on VO_2_max, an experimental model, where arm exercise is added to leg exercise can be used (Bergh et al., 1976).

The present study aimed to investigate the effects of adding arm exercise to leg exercise in individuals with CKD, an exercise model that, to our knowledge, has not previously been used to study limiting factors for aerobic exercise capacity in CKD. Our main hypothesis was that by increasing the active muscle mass, VO2peak would increase more in the CKD group than in healthy controls.

## 2 MATERIALS AND METHODS

### 2.1 Study population

Ten individuals with non‐dialysis CKD stages 3–5, aged 28–59 years, four females and six males (CKD), and 10 healthy controls were included. The control group was matched to the CKD group for age, sex, body size, and physical activity level. The inclusion criterion for the CKD group was non‐dialysis CKD stages 3–5. The inclusion criteria for the controls were absence of kidney disease, diabetes, or cardiovascular disease. The exclusion criteria for all participants were current malignancy and kidney transplantation/donation. Further exclusion criteria for the CKD group were diabetes and kidney disease secondary to diabetes or cardiac disease.

Recruitment took place through renal medicine outpatient clinics in the Stockholm area and an advertisement on the website of Karolinska Institutet. Participants were examined by a medical doctor and included in the study provided they fulfilled the inclusion criteria.

The study was reviewed and approved by the Swedish Ethical Review Authority (nr 2021–05718‐01). Written informed consent was obtained from all participants.

At the inclusion visit, the following data were collected: weight, height, resting blood pressure, 12‐lead electrocardiogram (ECG), rectus femoris muscle thickness, leg muscle strength, and cardiac structure and function. The participants also rated their physical activity level according to the Saltin–Grimby Physical Activity Level Scale (Saltin & Grimby, 1968). A maximal leg cycling test with ECG was performed to assess their fitness level as well as a familiarization session with combined leg and arm exercise.

### 2.2 Experimental design and cardiopulmonary exercise test

On test days 1 and 2, the participants were randomized to start with either a maximal exercise test with legs on an electronically braked cycle ergometer (Monark LC6; Vansbro, Sweden) or a maximal exercise test where arm cycling (Rodby Innovation AB; Vänge, Sweden) was added during the leg exercise test. All tests were driven to volitional exhaustion, and all subjects performed both tests on separate occasions. Heart rate and workload were measured continuously. Capillary blood lactate was measured at rest, during warm‐up, at peak exercise, and 3–5 min post‐exercise. Heart rate was measured with both ECG (Quark Stress Testing ECG; COSMED, Rome, Italy) and a chest strap (Garmin HRM‐Run; Olathe, KS, USA). If there were ECG artifacts at maximal exercise, the heart rate from the chest strap was used in both tests. The ECG data were used in all but five subjects. Both tests started with a 5‐min warm‐up at a workload of 50 W. Oxygen uptake (VO_2_) was determined continuously using an online gas analyser (Quark CPET; COSMED) that measured fractions of inspired and expired O_2_ and CO_2_ and recorded breath‐by‐breath values. VO_2_peak was defined as the highest 30‐s average reached during the test. Criteria for a maximal test were either a plateau in VO_2_ or a respiratory exchange ratio (RER) ≥1.1 and a rating of perceived exertion (RPE) ≥18 on the Borg RPE (rating of perceived exertion) scale (Borg, 1982). Peak ventilation (VEmax) and breathing reserve $\left((\mathrm{FEV}1\times 40-\text{VEmax}\right)/\left(\mathrm{FEV}1\times 40\right))\times \;100$ were determined as the highest 20‐s average interval. Dynamic spirometry (Quark PFT; COSMED) was measured before exercise on test day 1. Criteria for disengagement was a cadence of <50 revolutions per minute for either legs or arms.

**FIGURE 1:**
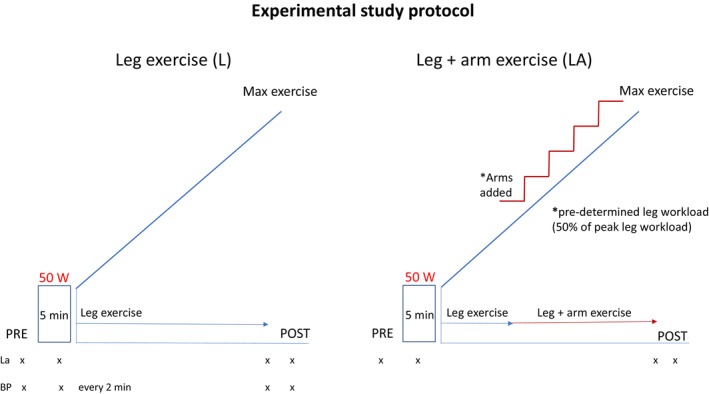
Experimental study protocol including two exercise tests: leg exercise (L) and combined leg and arm exercise (LA). Arm exercise was added at a pre‐determined leg workload of 50% of peak leg workload at inclusion. During L, systolic blood pressure (BP) was measured every 2–3 min, near max and at 3–4 min post‐exercise. BP was not measured during LA. Lactate (La) was analysed at rest, during warm‐up, at peak exercise, and at 3–5 min post‐exercise during L and LA. Heart rate was registered from 3 min pre‐ to 3 min post‐exercise with an electrocardiogram and chest strap.

The leg exercise test and combined leg and arm exercise tests were performed at similar times during the day (±1 h). All subjects were instructed to eat a similar meal before both tests and to refrain from caffeine intake or a larger meal at least 2 h before the test. The subjects were also told to refrain from intense physical activity within 48 h before test days. The experimental protocol is shown in Figure 1, and the exercise tests are described below.

### 2.3 Leg exercise test (L exercise)

Workload increased in a continuous ramp from 50 W with an increase of 10, 15, or 20 W per minute, based on the fitness of the subject, with the aim to achieve a test time of around 10 min. Systolic blood pressure was measured with a sphygmomanometer using the Doppler method.

### 2.4 Combined leg and arm exercise test (LA exercise)

**FIGURE 2:**
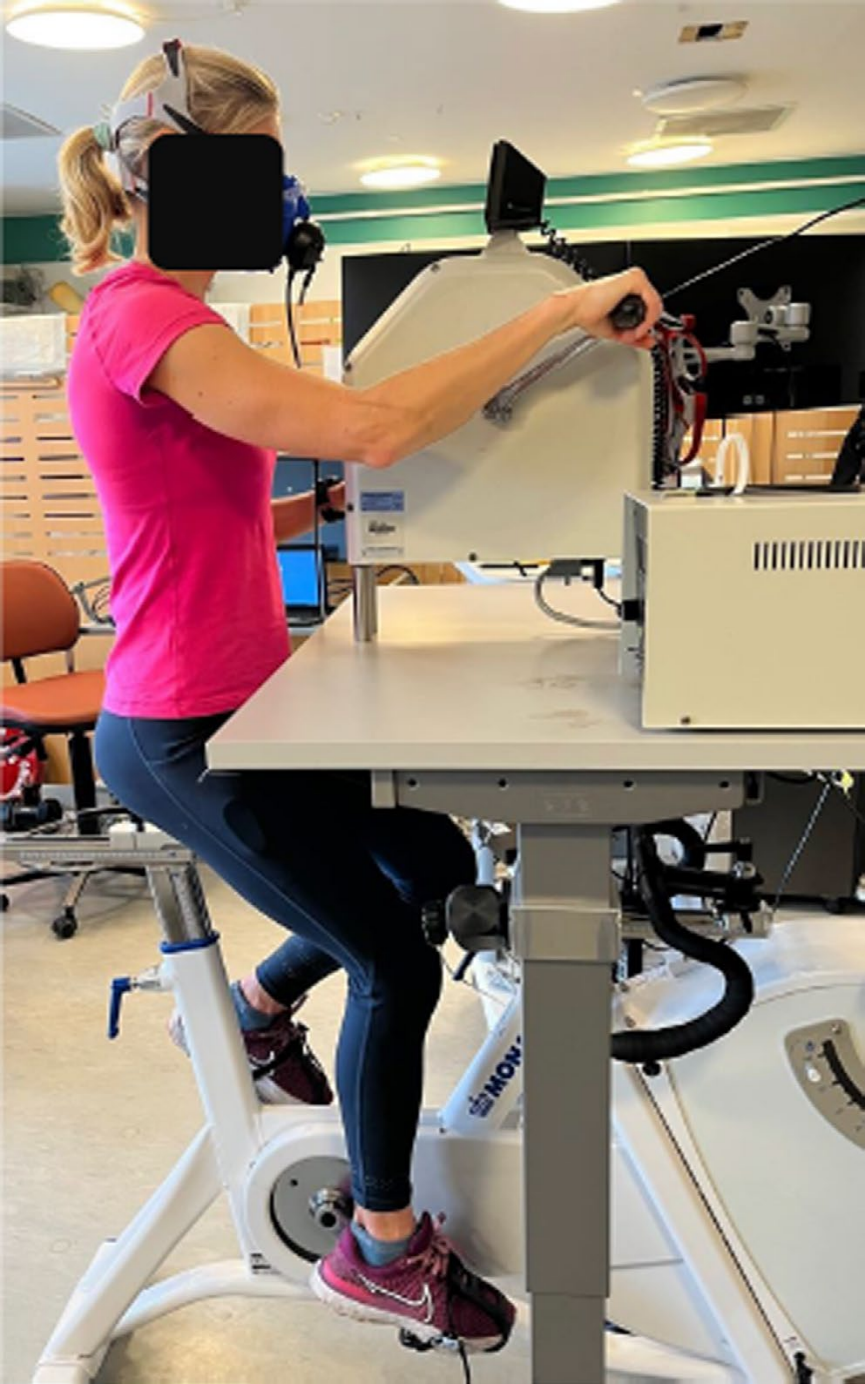
Combined leg and arm exercise set‐up.

The arm cycle was mounted on a specially built table that was adjusted in height for each subject to achieve an alignment between the ergometer's crankshaft and the glenohumeral joint. Further minor adjustments were made depending on individual preference. An image of the test setup is shown in Figure 2.

An incremental exercise test, using the same ramp as in the L exercise, was performed until the participant reached approximately 50% of maximal workload achieved at the exercise test during the inclusion visit. Thereafter, incremental arm work was added while the participants continued to cycle with their legs. This protocol was chosen with the goal of achieving a ratio of arm workload/total workload of 20%–25%, which generated the highest VO_2_ in a previous study on combined leg and arm exercise (Bergh et al., 1976).

The initial arm load for all participants was 10 W and increased in steps every minute. The step increase in arm load was half the increase in leg workload per minute. Arm workload at peak exercise was calculated according to the following formula: Arm workload at the highest completed step—((seconds at peak arm workload/60) × (step increase per minute)). Arterial pressure was not measured during the LA exercise.

The reliability of the exercise tests was assessed in a pilot study that included seven healthy subjects (age range, 50–67 years). The subjects came to the laboratory on two separate occasions, separated by a minimum of 2 days, and performed L and LA exercise in a randomized order on each occasion, according to the same protocol as in the present study. The coefficients of variation for VO_2_peak were approximately 5% for both the L and LA exercise (unpublished data: Ragad Said, Helena Wallin, Anette Rickenlund, and Eva Jansson).

### 2.5 Muscle thickness and strength

Rectus femoris thickness was measured by ultrasound (Vivid s70; General Electric Healthcare, Chicago, IL, USA) using a linear transducer (8 mHz) as a proxy for skeletal muscle mass. Ultrasound‐measured rectus femoris thickness has shown a strong correlation to muscle thickness and cross‐sectional area measured by computed tomography and magnetic resonance imaging (Giles et al., 2015; Sahathevan et al., 2021). Subjects were examined in a supine position with their knees in full extension. An ultrasound transducer was placed transversally to the thigh, with the transducer oriented at a 90° angle to the muscle bundles. An ample amount of water‐soluble transmission gel was applied. The scanning site was located at 2/3 (distal, closer to the knee) of the distance between the anterior superior iliac spine and the superior pole of the patella. The average value of three consecutive measurements was reported as the rectus femoris thickness.

Leg muscle strength was determined by measuring isokinetic and isometric peak torque for the knee extensors using isokinetic dynamometry (Biodex System 4 Pro; Medical Systems, Shirley, NY, USA). Peak isometric and isokinetic torque (60°/s and 180°/s) were measured for both the right and left legs, and the average value of both legs was reported. All subjects performed three maximal repetitions for each leg at each angular velocity, with a 30‐s rest period employed between trials. The isometric test was performed at a fixed knee angle of 120° (i.e., 60° from full extension), and the participants were instructed to apply as much force as possible for 5 s. Three trials were administered for each leg with 30 s of recovery between each trial.

### 2.6 Cardiac structure and function

Echocardiography was performed according to current guidelines (Lang et al., 2015) using the Vivid s70 ultrasound machine (General Electric Healthcare, Chicago, IL, USA). Stroke volume was calculated by multiplying the left ventricular (LV) outflow tract (LVOT) area with the LVOT velocity–time integral. LV volumes and ejection fraction were calculated using the bi‐plane Simpson method. LV diameter and tricuspid annular plane systolic excursion (TAPSE) were measured according to guidelines. LV mass was calculated according to the Deveraux cube formula (Devereux et al., 1986; Lang et al., 2015).

### 2.7 Biochemical analyses

Capillary lactate concentration was measured using Lactate Scout 4 (EKF Diagnostics, Cardiff, UK, catalogue number for strips 7023–3405‐0846) Hb concentration was determined via venous blood sampling before the second exercise test. Additionally, capillary Hb was measured in seven of the 10 CKD subjects before each exercise test. There were no systematic differences between capillary and venous Hb, and the difference between test days was less than 5 g/L for all subjects. Plasma creatinine and cystatin C were measured before the second exercise test at the Laboratory Medicine Study Center at Karolinska University Hospital with a Cobas 8000 c701 (Basel, Switzerland) modular analyser using an enzymatic, photometric method (creatinine) and a latex‐enhanced immunoturbidimetric method (cystatin‐C). In some instances, plasma values were obtained through medical records. The estimated glomerular filtration rate (eGFR) was calculated using the Chronic Kidney Disease‐Epidemiology Collaboration equation (Levey et al., 2009).

### 2.8 Statistical analysis and calculations

There are no previous studies on combined arm and leg cycling in CKD. However, based on previous data from our group, we calculated that a study population of 9–14 individuals would be sufficient to detect a difference in exercise capacity and peak heart rate (with 80% power and *α* = 0.05) between individuals with CKD 4–5 and healthy controls. In previous studies measuring leg blood flow during exercise, a study population of 6–10 individuals has been sufficient to detect a difference compared to a control group (Bradley et al., 1990; Marrades et al., 1996). Therefore, we aimed for a study population of 10 individuals in each group. All data are presented as mean ± standard deviation (SD) unless otherwise stated. To assess differences in basal characteristics between the CKD and control groups, Student's *t*‐test was used, except for categorical variables, where the Mann–Whitney *U* test was employed. To determine whether the difference between the L and LA exercise differed between CKD and controls, a repeated‐measures analysis of variance was performed. If there was a significant interaction between groups (CKD or controls) and tests (L or LA exercise), the difference between the two groups was considered significant. Student's *t*‐test (paired or unpaired) was used to further describe these interactions without corrections for multiple testing. The Pearson correlation coefficient (r) and linear regression were used to analyse the linear relationship between variables. Statistical significance was defined as a *p*‐value <0.05 for a two‐tailed test. IBM SPSS Statistics was used for the analyses.

## 3 RESULTS

### 3.1 Subject characteristics

Anthropometric, biochemical, cardiac and lung function characteristics, as well as physical activity level, for subjects with CKD (*n* = 10) and controls (*n* = 10) are shown in Table 1. The CKD group covered non‐dialysis stages 3a–5 (two in stage 3a, one in stage 3b, five in stage 4, and two in stage 5). The etiology of CKD included IgA nephropathy (*n* = 6), Alports nephropathy (*n* = 1), Lupus nephritis (*n* = 1), atypical hemolytic uremic syndrome (*n* = 1), and one case where the etiology was unknown. A successful matching yielded no significant differences in sex, age, height, weight, or physical activity level between CKD and controls. Hb level was significantly lower in CKD than in controls. The echocardiographic exam showed no evidence of systolic or diastolic dysfunction, cardiomyopathy, or valvular disease in any of the subjects. However, LV mass was significantly higher in CKD than in controls. Lung function and resting blood pressure did not differ between groups. No subject had symptoms or electrocardiographic evidence of myocardial ischaemia or arrythmia during exercise testing.

**TABLE 1:** Study group characteristics.

Variables	CKD (*n* = 10)	Controls (*n* = 10)	*p*‐Value
Age (years)	48 ± 11	49 ± 11	0.9
Male, *n* (%)	6 (60)	6 (60)	0.9
Height (cm)	174 ± 8	174 ± 13	0.9
Weight (kg)	79 ± 18	79 ± 13	0.9
Body mass index (kg/m^2^)	25.8 ± 3.9	26.1 ± 4.2	0.9
Body surface area (m^2^)	1.94 ± 0.25	1.94 ± 0.21	0.9
Systolic blood pressure (mmHg)	120 ± 37	132 ± 14	0.4
Diastolic blood pressure (mmHg)	82 ± 7	82 ± 10	0.9
Heart rate rest (beats/min)	65 ± 7	61 ± 11	0.4
Physical activity level^a^	3 (2.75–4)	3 (3–3.25)	0.9
Biochemical analyses			
Hemoglobin (g/L)	115 ± 19	143 ± 12	0.001
Creatinine (mmol/L)	288 ± 130	80 ± 11	<0.001
eGFR (mL/min/1.73 m^2^)	26 ± 17	90 ± 16	<0.01
Cystatin C (mg/L)	2.75 ± 1.09	0.77 ± 0.08	<0.001
Cardiac function			
LVEDD (cm)	5.2 ± 5	4.9 ± 6	0.2
LVEDVI (mL/m^2^)	62 ± 11	54 ± 9	0.1
Stroke volume index (mL/m^2^)	50 ± 10	44 ± 8	0.2
LVMI (g/m^2^)	98 ± 21	77 ± 16	0.03
LV ejection fraction, EF (%)	57 ± 17	62 ± 5	0.4
LV diastolic function, E/é	7.5 ± 1.2	6.6 ± 1.5	0.1
RV systolic function, TAPSE (cm)	2.7 ± 3	2.8 ± 3	0.9
Lung function			
FEV1 (L)	3.4 ± 0.7	3.4 ± 0.7	0.9
FVC (L)	4.2 ± 0.8	4.4 ± 1.0	0.8
Medications, *n*			
Erythropoietin	3	0	
Iron supplementation	6	0	
ACE inhibitor	4	0	
Angiotensin II blockers	5	0	
Other medication for hypertension	9	0	
Lipid‐lowering medication	6	0	
Other chronic medication	9	5	

*Note*: Values are reported as number (percentage) or mean ± standard deviation or median (interquartile range) for categorical variables.

Abbreviations: CKD, chronic kidney disease; E/é, early filling velocity/early diastolic myocardial velocity; EF, ejection fraction; eGFR, estimated glomerular filtration rate; FEV1, forced expiratory volume in 1 second; FVC, forced vital capacity; LV, left ventricle; LVEDD, left ventricular end‐diastolic diameter; LVEDVI, left ventricular end‐diastolic volume index; LVMI, left ventricular mass index; RV, right ventricle; TAPSE, tricuspid annular plane systolic excursion.

^a^ Physical activity level was rated according to the Saltin–Grimby Physical Activity Level Scale, where 4 is the most active.

Current medications for both groups are listed in Table 1. One patient in the CKD group was on beta blocker therapy, which was withdrawn 8 days before test day 1. The second test for that subject was performed 3 days later, while the beta blocker was still on hold. Three subjects in the CKD group were on erythropoietin therapy. Their tests were scheduled so that the interval was the same between erythropoietin injections and the L and the LA exercise, respectively. Medicines in the controls included beta‐blocking eye drops (*n* = 2), antidepressants/selective serotonin reuptake inhibitors (*n* = 1), hormone replacement therapy/estrogen, and progestogen (*n* = 1) and a hormonal intrauterine device (*n* = 1).

### 3.2 Response to leg exercise and combined leg and arm exercise

Six subjects in each group started with the L test, and four subjects in each group started with the LA exercise. The number of days between tests was 10 ± 8 (range, 3–42 days) in CKD and 11 ± 11 (range, 2–28 days) in controls. Peak exercise response to L and LA exercise is shown in Table 2, and workloads and exercise time with arm exercise are shown in Table 3.

**TABLE 2:** Response to leg exercise and combined leg and arm exercise.

	CKD (*n* = 10)		Controls (*n* = 10)		*p*‐Value		
	Leg (L)	Leg + arm (LA)	Leg	Leg + arm	ANOVA interaction test & group	LA vs. L (CKD/controls)^a^	CKD vs. controls (L/LA)^b^
VO_2_ peak (L/min)	2.45 ± 0.77	2.65 ± 0.9	2.82 ± 0.70	2.84 ± 0.69	0.02	0.007/0.7	0.3/0.6
VO_2_ peak (mL/(kg·min))	30.7 ± 5.8	33.1 ± 6.9	35.8 ± 7.2	36.1 ± 7.2	0.01	0.003/0.8	0.1/0.4
Peak heart rate (beats/min)	164 ± 17	167 ± 14	172 ± 15	172 ± 10	0.2	0.1/0.8	0.3/0.4
Predicted peak heart rate (%)	96 ± 9	97 ± 8	101 ± 9	100 ± 6	0.2	0.1/0.8	0.2/0.3
Peak RER	1.20 ± 0.06	1.18 ± 0.08	1.20 ± 0.06	1.17 ± 0.04	0.7	0.18/0.09	0.9/0.7
Peak RPE	19.4 ± 0.6	19.3 ± 0.9	19.7 ± 0.5	19.1 ± 0.1	0.2	0.9/0.04	0.3/0.5
Peak lactate (mmol/L)	10.0 ± 3.5	9.4 ± 2.9	10.4 ± 3.2	9.1 ± 2.4	0.2	0.4/0.1	0.8/0.8
Exercise time (min)	9:55 ± 2:20	8:51 ± 2:43	11:07 ± 1:45	9:40 ± 1:35	0.2	<0.001/<0.001	0.2/0.4
Peak leg workload (W)	211 ± 74	195 ± 76*	247 ± 58	221 ± 55	0.002	<0.001/<0.001	0.2/0.4
Peak leg workload/kg	2.64 ± 0.65	2.42 ± 0.67	3.15 ± 0.65	2.82 ± 0.61	0.01	<0.001/<0.001	0.09/0.2
Total workload (W)	211 ± 74	244 ± 94	247 ± 58	274 ± 66	0.4	<0.001/<0.001	0.2/0.4
Peak VE (L/min)	114 ± 34	123 ± 42	128 ± 27	126 ± 25	0.1	0.1/0.6	0.3/0.8
Breathing reserve (%)	18 ± 14	12 ± 17	5 ± 10	7 ± 12	0.05	0.1/0.5	0.03/0.5

*Note*: Predicted peak heart rate is based on the formula 220‐age. *p < 0.05.

Abbreviations: CKD, chronic kidney disease; HR, heart rate; L, leg exercise; LA, combined leg and arm exercise; RER, respiratory exchange ratio; RPE, rating of perceived exertion; VE, minute ventilation.

^a^ Paired *t*‐test.

^b^ Unpaired *t*‐test.

**TABLE 3:** Workloads and exercise time with arm exercise.

	CKD (*n* = 10)	Controls (*n* = 10)	*p*‐Value
Peak workload arms (W)	49 ± 18	53 ± 12	0.6
Arm workload/total workload (%)	20.3 ± 0.6	19.2 ± 1.2	0.03
Leg workload when adding arm exercise (W)	105 ± 37	122 ± 29	0.3
Time with arm exercise (min)	5:49 ± 1:16	5:40 ± 0:43	0.8

Abbreviations: CKD, chronic kidney disease.

VO_2_peak was significantly higher in the LA than in the L exercise in CKD, while no significant difference in VO_2_peak was found between LA and L for controls (Table 2). The difference in VO_2_peak in LA compared with L was significantly higher in CKD (0.20 ± 0.18 L/min or 2.31 ± 1.78 mL/(kg·min)) than in controls (0.019 ± 0.12 L/min or 0.26 ± 1.62 mL/(kg·min); *p* = 0.02 and 0.01, respectively, for the difference between groups). This corresponds to a 7% increase in VO_2_ between LA and L in CKD and a non‐significant increase of 1% in controls. There was no significant difference between the two study groups in VO_2_peak, peak workload, and peak heart rate, either for the L or the LA exercise, although there was a trend toward higher values in the controls in all these variables. Peak heart rate was not significantly higher in LA compared with L in either CKD or controls, even though there was a trend toward higher peak heart rate in CKD in the combined leg and arm exercise (*p* = 0.1).

In both groups, leg peak workload was lower in the LA than in the L test, while total workload (legs + arms) was higher in the LA than in the L test. The difference in leg peak workload between LA and L was significantly higher for controls than for CKD, both in absolute terms (*p* = 0.002) and relative to body weight (*p* = 0.01).

**FIGURE 3:**
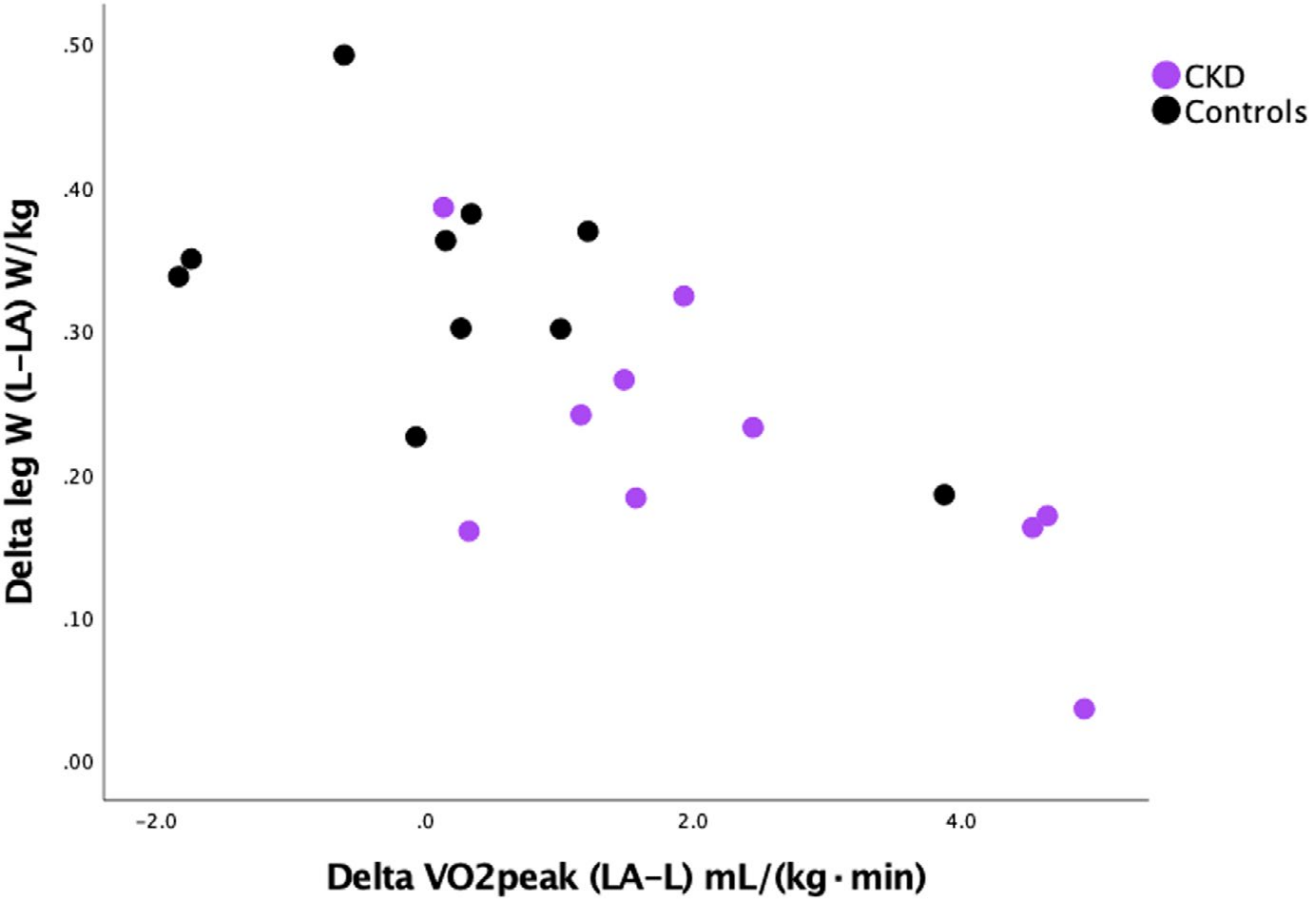
Relationship between the difference in leg peak workload (L – LA) and the difference in VO_2_peak (LA – L). L, leg exercise; LA, combined leg and arm exercise. *R* ^2^ = 0.52.

In a multiple linear regression analysis with the change in leg peak workload/kg (L – LA) as the dependent variable and the change in VO_2_peak in mL/(kg·min) (LA – L) and group (CKD or controls) as the independent variables, only the variation in increase in VO_2_peak in mL/(kg·min) contributed significantly to explain the variation in decrease in leg peak workload/kg. The interaction term group * increase in VO_2_peak in mL/(kg·min) (LA – L) was not significant. This means that all subjects followed the same regression line independent of group (Figure 3). In addition, 56% of the variation in decrease in leg peak workload was explained by the variation in increase in VO_2_peak.

### 3.3 Criteria for maximal tests

Peak RER, peak RPE, and peak lactate reached high values in both groups and in both tests and did not differ significantly between CKD and controls. Peak RER and peak lactate did not differ between the LA and L tests in either group, whereas peak RPE was significantly lower in the L than in the LA test in controls. Breathing reserve was significantly higher in CKD compared with controls in the L test, but not in the LA test. Although breathing reserve tended to be lower in LA than in L in CKD, the difference was not statistically significant (*p* = 0.1). Peak systolic blood pressure during leg exercise reached similar values in both groups: 192 ± 26 mm Hg in CKD and 195 ± 29 mm Hg in controls (*p* = 0.2).

All subjects reached objective criteria for a maximal test (plateau in VO_2_ or RER ≥1.1). All but two subjects also fulfilled the subjective criteria (RPE rating ≥18). One subject in the CKD group gave a maximal RPE rating of 17 at the LA test and cited knee pain as a limiting factor. That subject reached an RER of ≥1.1 (similar to the L test). One subject in the control group rated peak RPE at 17 at the LA test but fulfilled the objective criteria for a maximal test. The difference in VO_2_peak (LA – L) between the groups remained significant (*p* = 0.009) even when excluding these two subjects from the analysis.

### 3.4 Leg muscle thickness and strength

Strength data and rectus femoris thickness are displayed in Table 4. Even though absolute values were lower in CKD, neither isokinetic nor isometric strength were significantly different between groups. Rectus femoris thickness showed a similar trend and did not differ significantly between CKD and controls.

**TABLE 4:** Leg muscle strength and rectus femoris muscle thickness.

	CKD (*n* = 10)	Controls (*n* = 10)	*p*‐Value
Leg muscle strength			
Isokinetic peak torque 60° (Nm)	184 ± 71	197 ± 54	0.7
Isokinetic peak torque 180° (Nm)	127 ± 54	142 ± 39	0.5
Isometric peak torque (Nm)	209 ± 88	217 ± 51	0.8
Isokinetic peak torque 60° per kg (Nm/kg)	2.29 ± 0.63	2.51 ± 0.61	0.5
Isokinetic peak torque 180° per kg (Nm/kg)	1.57 ± 0.46	1.81 ± 0.45	0.3
Isometric peak torque per kg (Nm/kg)	2.60 ± 0.76	2.78 ± 0.65	0.6
Leg muscle thickness			
Rectus femoris mean left and right (cm)	1.78 ± 0.53	2.00 ± 0.32	0.3
Rectus femoris left (cm)	1.75 ± 0.53	1.97 ± 0.32	0.3
Rectus femoris right (cm)	1.82 ± 0.53	2.10 ± 0.31	0.2

*Note*: Strength data are expressed as mean value of left and right leg.

Abbreviations: CKD, chronic kidney disease.

## 4 DISCUSSION

### 4.1 Main findings

The study design, by which additional muscle mass was added by combined leg and arm exercise, aimed to elucidate whether subjects with CKD had a peripheral or muscular limitation, rather than a central limitation to VO_2_max. Our results show that by increasing the active muscle mass, VO_2_peak increases in a group of non‐dialysis CKD subjects. This differed significantly from healthy controls, in whom VO_2_peak did not increase. The decrease in breathing reserve in the LA compared with the L test further supports the observation that the CKD group did not exhaust their cardiopulmonary reserve in the L test. In addition, peak leg workload decreased more when arm exercise was added in the controls than in CKD. These findings support the hypothesis of a peripheral muscular limitation to VO_2_max in CKD.

Although our data suggest a peripheral limitation in CKD, we did not study the mechanisms behind this. Other studies on CKD have found support for several peripheral mechanisms that may impair oxygen delivery. These include an inadequate skeletal muscle vasodilatory response to exercise (Sprick et al., 2019), a low capillary density (Lewis et al., 2012; Moore, Parsons, et al., 1993) or reduced oxygen flow from the muscle capillaries to the mitochondria (O_2_ conductance) (Marrades et al., 1996; Sala et al., 2001) due to, for example, a thickened capillary membrane (Stray‐Gundersen et al., 2016). As the last step in the oxygen transport and utilization chain, mitochondrial dysfunction (Gamboa et al., 2016, 2020; Kestenbaum et al., 2020; Roshanravan et al., 2016) may further contribute to the reduced oxygen uptake in CKD. Recent advances in muscle microcirculation research also highlight several limiting factors other than diffusion distance, such as red blood cell flux, longitudinal capillary recruitment, and capillary three‐dimensional geometry (Poole & Edward, 2019; Poole & Musch, 2023). To our knowledge, these mechanisms have not yet been investigated in CKD. Of note, anemia could possibly influence both convective and diffusive oxygen transport in CKD. Previous research has shown that anemic patients with CKD lack the expected compensatory response to exercise, which would include higher cardiac output and higher peripheral oxygen extraction (Macdonald et al., 2012; Marrades et al., 1996). Furthermore, in a recent study on male non‐dialysis CKD patients (Chinnappa et al., 2021), the arterio‐venous oxygen difference (a‐vO_2_ diff) was found to be a stronger predictor of VO_2_peak than peak stroke volume and peak heart rate. In that study, Hb contributed significantly to the variation in a‐vO_2_ diff. While cardiovascular and autonomic dysfunction is common in CKD and may influence systemic oxygen delivery (Howden et al., 2015; Park & Middlekauff, 2015; Van Craenenbroeck et al., 2016; Wallin et al., 2018), and thereby exercise capacity, our study, together with previously mentioned research (Chinnappa et al., 2021; Marrades et al., 1996; Moore, Parsons, et al., 1993; Sala et al., 2001; Stray‐Gundersen et al., 2016), lends support to the concept of peripheral factors limiting VO_2_max in CKD.

### 4.2 Peak leg workload decreases in combined leg and arm exercise

The decrease in peak leg workload between the L and LA tests was significantly larger in the controls than in CKD. Furthermore, the decrease in peak leg workload was negatively correlated with the increase in VO_2_ between the L and LA tests. This indicates that subjects who could not increase VO_2_max when arm exercise was added had a larger decrease in peak leg workload between tests compared with those who could increase VO_2_max. Exercise time was shorter in combined exercise, as would be expected since the same leg exercise protocol was used in both tests. But there were no differences between groups regarding the difference in exercise time between the two tests. Competition for oxygen supply between the arms and legs might explain why peak leg workload was lower in combined exercise and more so in subjects who reached or were already close to VO_2_ max with leg exercise. On this topic, Secher et al. (Secher et al., 1977) demonstrated that, beyond a certain work intensity, exercise in one muscle group could influence the oxygen uptake in another active muscle group. They reported reduced leg blood flow and leg oxygen uptake when adding arm exercise to high‐intensity leg exercise and suggested that the increased sympathetic activity caused by the additional arm work may counteract metabolic vasodilation and mediate vasoconstriction in exercising muscle (Secher et al., 1977). These mechanisms could explain why, in healthy subjects, VO_2_max is generally only mildly elevated at most when arm exercise is added to exercise performed by large muscle groups such as the legs.

### 4.3 Comparison with other studies on combined leg and arm exercise

To the best of our knowledge, only one previous study has investigated combined leg and arm exercise on patient groups. Jondea et al. (Jondeau et al., 1992) added arm exercise to leg exercise in individuals with heart failure and found that both VO_2_peak and peak heart rate increased compared with controls, who did not increase their VO_2_peak. Our present findings in the controls agree with previously published data, which show a slight increase at most in VO_2_max with combined leg and arm exercise (Astrand & Saltin, 1961; Bergh et al., 1976; Jondeau et al., 1992; Stenberg et al., 1967). This is consistent with a central or cardiac limitation. These previous studies include early studies from Åstrand (Astrand & Saltin, 1961) and Stenberg et al. (Stenberg et al., 1967), who reported no increase in VO_2_max with combined leg and arm exercise compared with leg exercise only in young healthy adults. Bergh et al. (Bergh et al., 1976) reported a 5% increase in VO_2_max with combined work compared with leg cycling in 10 healthy men, with the greatest increase observed when the proportion of arm work to total workload was 20%. However, with a proportion of arm work to total workload of 40%, however, there was a risk that the subjects would stop exercising due to exhaustion of arm muscles (Bergh et al., 1976).

### 4.4 Heart rate response

A reduced peak heart rate is a well‐known feature in individuals with CKD, and peak heart rate is an important determinant of VO_2_max in this group (Chinnappa et al., 2018, 2021; Howden et al., 2015; Moore, Brinker, et al., 1993; Wallin et al., 2018). Cardiac autonomic dysfunction, including decreased catecholamine sensitivity, is the most common explanation behind the blunted chronotropic response in CKD (Clyne et al., 2016; Hathaway et al., 1998; Kettner et al., 1984; Ranpuria et al., 2008). To our knowledge, this is the first study to investigate the effect of active skeletal muscle mass on peak heart rate in CKD. The difference in peak heart rate in combined exercise compared with leg exercise only was not statistically significant, even though a tendency toward higher values in the combined test was noted (164 beats/min vs. 167 beats/min; *p* = 0.1). Peak heart rate VO_2_peak was not significantly different between CKD and controls, although there was a tendency toward lower values in the CKD group. Most previous studies (Astrand & Saltin, 1961; Bergh et al., 1976; Gleser et al., 1974; Secher et al., 1974; Stenberg et al., 1967) on healthy subjects have not reported a significantly higher peak heart rate in combined leg and arm exercise compared with leg exercise only. This contrasts with the study in heart failure, where the peak heart rate increased in combined exercise (Jondeau et al., 1992). However, these subjects with heart failure had a lower peak heart rate during leg exercise than the subjects with CKD in the present study.

### 4.5 Muscle thickness and strength

A loss of muscle mass and strength is often reported in CKD, being more pronounced in patients on dialysis (Beetham et al., 2015; Diesel et al., 1990; Hiraki et al., 2013; Isoyama et al., 2014). Although no significant differences in leg muscle strength or muscle thickness were found between CKD and controls in the present study, a trend toward lower values was observed in the CKD group. As previously mentioned, the CKD group was physically active without a significantly reduced VO_2_peak. It may be speculated that while these subjects do not show evidence of muscle weakness or atrophy, structural and microcirculatory changes in the skeletal muscle might be present.

### 4.6 Strengths and study limitations

An important strength of the present study is the control group, which was tightly matched with the CKD group in terms of age, sex, body size, and physical activity level. Moreover, there were no major comorbidities such as diabetes, cardiovascular disease, or respiratory disease that could have had an influence on exercise capacity and thereby confound our results. In addition, the CKD group was tested without beta blockers, a medication common in this patient group and a drug that adds a central limitation which could have masked the peripheral limitation to VO_2_max.

This study has some limitations. First, the sample size was quite small. Although it was large enough to prove robust differences, such as the increased VO_2_peak in the LA compared with the L test in the CKD group, evaluating trends in other variables might have benefited from a larger sample size. Second, the individuals with CKD represent a group of relatively physically active patients, but in a less active group of patients, the differences in VO_2_peak, peak heart rate, and muscular measurements might have been more pronounced. Despite this, we found a significant difference between groups regarding the change in VO_2_peak between leg and combined leg and arm exercise. Third, as the study was not blinded, it is also important to address the potential bias of the test leader. RPE in the control subjects was significantly lower for the LA than for the L test. A slightly lower RPE rating could be due to the subjects being unaccustomed to the combined leg and arm exercise compared with the leg exercise only, although that would apply to the CKD group as well. As all tests were driven to exhaustion with high RER values and/or a VO_2_ plateau, indicating maximal tests, as well as high RPE ratings and low breathing reserves, we do not believe that a potential submaximal effort has influenced our findings. No VO_2_ max verification trial was performed, which would have strengthened our results, but we have several criteria for maximal tests that did not differ between the two exercise tests. The subjects were also randomized to start with either of the tests. The same work rate increments for the legs were used in both the combined exercise and the leg exercise; however the increase in work rate for the arms was in one‐minute steps and not continuous. It is difficult to predict how this influenced the results.

Lastly, we did not do invasive measurement such as cardiac output and leg muscle blood flow measurements, which would have provided more definitive evidence of exercise limitation. However, these measurements are challenging in a clinical population.

## 5 CONCLUSION

VO_2_max in individuals with CKD is dependent on the active muscle mass, supporting, but not confirming, the hypothesis of a peripheral limitation to VO_2_max in this patient group. By contrast, the healthy subjects appeared to have a more central limitation to VO_2_max. Although a comprehensive understanding of the pathophysiological processes contributing to muscle dysfunction in CKD may not have been attained yet, several potential mechanisms that could be further explored in future research exist. When measuring aerobic exercise capacity in individuals with CKD, one should bear in mind that it is an integrative measure of cardiac, vascular, and muscular function, as well as anemic and possibly nutritional status, rather than a measurement of cardiac function. Our findings reinforce the importance of skeletal muscle function as a determinant of aerobic exercise capacity in CKD. Further studies, with measurements of cardiac output and arteriovenous oxygen difference during exercise, could provide more clear evidence of exercise limitation in this population.

## AUTHOR CONTRIBUTIONS

The experiments in this study were carried out at the Division of Clinical Physiology, Department of Laboratory Medicine at Karolinska Institutet. HW, EJ, PS, AR, and RS designed the present study. HW, PS, and EJ performed the analyses and interpretation of the data. HW, AR, PZ, and SL contributed to the data collection and patient recruitment. HW performed the statistical analyses and wrote the manuscript. PS and EJ supervised and revised the manuscript. All authors approved the final version of the manuscript and agree to be accountable for all aspects of the work in ensuring that questions related to the accuracy or integrity of any part of the work are appropriately investigated and resolved. All persons designated as authors qualify for authorship, and all those who qualify for authorship are listed.

## FUNDING INFORMATION

The study was supported by grants from the Stockholm County Council (grant numbers 2020 and 2021) received by MJE, grants from the Swedish Kidney Foundation (grant number F2017‐0048) received by AR and a grant from Lindhés advokatbyrå (grant number LA2018‐0216) received by HW. The funders had no role in the study design, data collection and analysis, decision to publish, or preparation of the manuscript. Open Access funding was provided by Karolinska Institutet.

## CONFLICT OF INTEREST STATEMENT

The authors declare no competing interests.

## ETHICS STATEMENT

The study was carried out in accordance with the WMA Declaration of Helsinki. An approval from the Swedish Ethical Review Authority (nr 2021‐05718‐01) was obtained before starting the study.

## Data Availability

The data supporting the findings are presented in the paper and the tabulated raw data are available upon request from the corresponding author.
